# Maternal cardiovascular and pregnancy outcomes among pregnant patients with mechanical and bioprosthetic valves

**DOI:** 10.1002/pmf2.70506

**Published:** 2026-11-14

**Authors:** Angela Essa, Lara C. Kovell, Gianna L. Wilkie

**Affiliations:** ^1^ Department of Obstetrics and Gynecology University of Massachusetts Chan—Baystate Medical Center Springfield Massachusetts USA; ^2^ Division of Cardiovascular Medicine Department of Medicine University of Massachusetts Chan School of Medicine Worcester Massachusetts USA; ^3^ Division of Maternal Fetal Medicine Department of Obstetrics and Gynecology University of Massachusetts Chan School of Medicine Worcester Massachusetts USA

**Keywords:** bioprosthetic valve, maternal outcome, mechanical valve, perinatal outcome, pregnancy

## Abstract

**Introduction:**

Patients with mechanical heart valves in pregnancy are at a substantially higher risk of adverse maternal and fetal outcomes. Management of patients with mechanical heart valves poses a unique challenge, and strategies for anticoagulation in these patients remain controversial. The goal of this project was to determine rates of adverse maternal and perinatal outcomes in patients with mechanical valves compared to those with bioprosthetic valves, and assess outcomes in patients with mechanical valves using low molecular weight heparin and warfarin.

**Methods:**

A retrospective query was performed using Epic Cosmos to evaluate pregnancies affected by mechanical and bioprosthetic heart valves between January 1, 2023 and January 1, 2026. Patient demographics and rates of adverse cardiac outcomes in the perinatal period, including heart failure, arrhythmia, and stroke, were assessed. The rate of anticoagulation with low molecular weight heparin and warfarin in patients with mechanical heart valves was evaluated, and cardiac and perinatal outcomes were determined and compared between groups. Rare outcomes were reported as <10 to protect patient privacy.

**Results:**

Baseline demographics between patients with mechanical and bioprosthetic heart valves were similar. Rates of heart failure (22.9%) and stroke (11.7%) were significantly higher in patients with mechanical compared to bioprosthetic heart valves, though the rates of maternal arrhythmia were similar between groups. Valve thrombosis was a rare outcome for patients with mechanical valves, with fewer than 10 events in the study sample. Perinatal outcomes including preterm and cesarean delivery were similar between groups.

**Conclusion:**

In conclusion, mechanical heart valves are associated with high rates of heart failure and stroke in pregnancy. Rates of adverse events may be similar for patients using low molecular weight heparin compared to warfarin for anticoagulation in pregnancy.

## 1 INTRODUCTION

Cardiovascular disease in pregnancy is increasing and represents a leading cause of severe maternal morbidity and mortality in the United States [1, 2]. The hemodynamic changes that occur and the hypercoagulable state that is induced during pregnancy can lead to increased risk of complications for patients with cardiac disease. Patients with mechanical heart valves are at particularly high risk of adverse outcomes, such as heart failure and thromboembolism [3].

While the incidence of mechanical heart valves among reproductive‐aged patients in the United States is not well‐established, there are approximately 100,000 valve replacements performed in the United States every year, with half of these being mechanical [4]. One population‐based study in the United Kingdom estimated the incidence of mechanical heart valves to be 3.7 per 100,000 pregnancies [5]. Another retrospective cohort study evaluating women ages 50 years or less in California, New Jersey, and New York State between 1990 and 2015 identified 11,930 women who underwent 14,017 valve replacements. Among these patients, there were 417 subsequent pregnancies, 52% of which were affected by mechanical heart valves [6].

While previous studies have sought to examine adverse maternal and fetal outcomes in patients with mechanical heart valves, research in this area remains limited, and existing studies include small numbers [3, 6, 7]. Additionally, limited data exist comparing outcomes in patients with mechanical heart valves who are using low molecular weight heparin (LMWH) versus warfarin for anticoagulation [8].

Therefore, the goals of this study were to determine maternal cardiovascular and pregnancy outcomes among patients with mechanical heart valves in pregnancy compared to those with bioprosthetic valves in pregnancy, and to further compare outcomes between patients using LMWH and warfarin for anticoagulation in the setting of a mechanical heart valve.

## 2 METHODS

This was a retrospective cohort study of pregnancies and patients obtained from the Epic Cosmos database, a dataset created in collaboration with multiple health systems that utilize Epic, and representing more than 300 million patient records from over 2067 hospitals from all 50 states and Washington, DC [9]. Participating healthcare systems contribute a limited dataset, which is centrally anonymized by Epic. This study was deemed exempt from requiring institutional review board approval given all data are deidentified. This study adheres to Strengthening the Reporting of Observational Studies in Epidemiology (STROBE) guidelines. Aggregate‐level data were collected for all demographic factors and outcome variables, as individual patient‐level data are not accessible through Epic Cosmos.

Charts in Epic Cosmos were queried for a diagnosis of a mechanical or bioprosthetic heart valve based on ICD‐10 codes, with these diagnoses present before or during pregnancy, between the dates of January 1, 2023 to January 1, 2026. ICD‐10 codes included Z95.3 for bioprosthetic heart valves and Z95.2 for mechanical heart valves. Pregnancies were then linked to patients to obtain demographic and maternal and perinatal outcome data for pregnancy episodes among patients with mechanical and bioprosthetic heart valves. Maternal demographics and maternal outcomes were evaluated for all patients who met criteria for inclusion in the study using aggregate‐level data. Perinatal outcomes were evaluated separately and accounted for all pregnancy episodes during the study period. Thus, if a patient had two pregnancy episodes during the study period, perinatal outcomes were evaluated twice. However, maternal demographics and maternal outcomes were only assessed once for each patient.

Demographic factors assessed included patient race, ethnicity, body mass index, census region, and smoking status. The presence of type 1 and type 2 diabetes mellitus (pregestational diabetes), chronic hypertension, and systemic lupus erythematosus were also assessed. Maternal cardiovascular and pregnancy outcomes evaluated included the development of a cardiac arrhythmia, heart failure, thromboembolism, valve thrombosis, endocarditis, myocardial infarction, stroke, preeclampsia, and maternal death. ICD‐10 codes used to assess for heart failure included I50.2x and I50.3x. ICD‐10 codes used to assess for stroke included I60.x, I61.x, I63.x, and I64.x. The ICD‐10 codes used to assess for thromboembolism included O88.211, O88.212, O88.213, O88.21, O88.22, and O88.23. The ICD‐10 codes used to determine rates of myocardial infarction included I21.x; for endocarditis I33.x and I38.x; for valve thrombosis T82.867; for arrhythmia I48.x, I49.x, and I47.x; and for preeclampsia O14.x.

Perinatal outcomes including preterm birth, gestational age at the time of delivery, mode of delivery, and pregnancy loss at <14 weeks’ gestation were also determined. Preterm birth was defined as any delivery between 20 weeks and prior to 37 weeks of gestation. Due to the design of the dataset, if <10 individuals were identified to have an outcome, the exact number was not provided to maintain patient confidentiality. All data were summarized with descriptive statistics and were compared for patients with a mechanical or bioprosthetic heart valve in pregnancy. Categorical variables were compared using Chi‐square tests, and continuous variables were compared using Student's *t*‐tests.

In addition to comparing maternal cardiovascular and perinatal outcomes between patients with mechanical and bioprosthetic valves, adverse cardiac and perinatal outcomes were determined for patients with mechanical heart valves who received LMWH or warfarin for anticoagulation in pregnancy. Outcomes were again compared between groups using Chi‐square tests for categorical variables and Student's *t*‐tests for continuous variables.

## 3 RESULTS

During the study period, we identified 384 patients with a mechanical heart valve experiencing 472 pregnancies, and 1127 patients with a bioprosthetic heart valve experiencing 1392 pregnancies.

Subjects in both the mechanical and bioprosthetic valve groups had a mean age of 32 years during the study period (Table 1). The majority of patients in both groups identified as White/Caucasian (68.5% in the mechanical valve group and 70% in the bioprosthetic valve group). There were no differences in race or ethnicity between groups. Average body mass index was slightly higher in the mechanical valve group (32.2 ± 9.4 kg/m^2^) compared to the bioprosthetic valve group (30.2 ± 4.8 kg/m^2^, *p *< 0.0001). Aspirin use was higher in the mechanical valve group compared to the bioprosthetic valve group (96.9% vs. 56.0%, *p* < 0.001). There was no difference in smoking status between groups, with the majority of patients being never smokers (Table 1).

**TABLE 1:** Demographics for patients with mechanical and bioprosthetic heart valves in pregnancy.

	Mechanical valve (*n* = 384)	Bioprosthetic valve (*n* = 1,127)	*p* value
Demographics			
Age (mean ± SD)	32 (5)	32 (6)	1.00
Race^a^			
Black/African American	69 (18.0%)	189 (16.8%)	0.58
White/Caucasian	263 (68.5%)	789 (70.0%)	
Asian	21 (5.5%)	47 (4.2%)	
Native Hawaiian/Pacific Islander	13 (3.4%)	27 (2.4%)	
Other	87 (22.7%)	225 (19.9%)
American Indian/Alaskan Native	<10	24 (2.1%)	
Unknown/None of the Above	19 (4.9%)	72 (6.4%)	
Ethnicity			
Hispanic	48 (12.5%)	121 (10.7%)	0.21
Non‐Hispanic	302 (78.6%)	873 (77.5%)	
Unknown	34 (8.9%)	133 (11.8%)	
Body Mass Index	32.2 (9.4)	30.2 (4.8)	**<0.0001**
Census Region			
South	158 (41.1%)	455 (40.4%)	0.79
Midwest	113 (29.4%)	317 (28.1%)	
Northeast	56 (14.6%)	161 (14.3%)
West	53 (13.8%)	179 (15.9%)	
Smoking Status			
Never	274 (71.4%)	810 (72.0%)	0.72
Former	73 (19.0%)	194 (17.2%)	
Current	26 (6.8%)	113 (10.0%)	
Aspirin use	372 (96.9%)	631(56.0%)	**<0.001**
Comorbidities			
Chronic hypertension	238 (62.0%)	361 (32.0%)	**<0.001**
Pregestational diabetes	40 (10.4%)	61 (5.4%)	**<0.001**
Systemic lupus erythematosus	<10	<10	–

*Note*: Numbers are *n* (%) or mean (standard deviation).

^a^ Patients can self‐report multiple races.

### 3.1 Maternal and perinatal outcomes

Maternal and perinatal outcomes for patients with mechanical and bioprosthetic heart valves are presented in Table 2. There was a significantly higher rate of heart failure in patients with mechanical heart valves compared to those with bioprosthetic valves (22.9% vs. 17.2%, *p* < 0.01). Maternal stroke was also higher in the mechanical valve group compared to the bioprosthetic valve group (11.7% vs. 5.2%, *p* < 0.0001), and there was a trend toward a higher rate of myocardial infarction in the mechanical valve compared to the bioprosthetic valve group, though this difference was not statistically significant (3.2% vs. 1.9%, *p* = 0.09). Rates of maternal arrhythmia and endocarditis were similar between groups, with 10.8% of patients in the mechanical valve group experiencing an arrhythmia and 14% experiencing endocarditis. Thromboembolism and valve thrombosis were rare outcomes in both groups, with valve thrombosis occurring in fewer than 10 patients in total in the mechanical heart valve group, consistent with an incidence of <0.03%. The incidence of preeclampsia was higher in the bioprosthetic valve group compared to the mechanical valve group (6.8% vs. 4%, *p* = 0.03).

**TABLE 2:** Maternal and perinatal outcomes in patients with mechanical versus bioprosthetic heart valves in pregnancy.

	Mechanical valve (*n* = 384)	Bioprosthetic valve (*n* = 1127)	*p* value
Maternal outcomes			
Arrhythmia	51 (10.8%)	172 (12.4%)	0.37
Heart failure	108 (22.9%)	240 (17.2%)	**<0.01**
Endocarditis	66 (14.0%)	231 (16.6%)	0.18
Thromboembolism	<10	36 (2.6%)	–
Valve thrombosis	<10	<10	–
Myocardial infarction	15 (3.2%)	26 (1.9%)	0.09
Stroke	55 (11.7%)	72 (5.2%)	**<0.0001**
Preeclampsia	19 (4.0%)	94 (6.8%)	**0.03**
Maternal death	<10	20 (1.8%)	–

	(*n* = 472)	(*n* = 1392)	
Perinatal outcomes			
Term birth	225 (47.7%)	727 (52.2%)	0.09
Preterm birth	96 (20.3%)	265 (19.0%)	0.54
Early pregnancy loss (<14 weeks)	67 (14.2%)	163 (11.7%)	0.15
Gestational age at delivery (weeks)	37 (2.9)	37.3 (2.9)	0.05
Cesarean delivery	143 (44.5%)	415 (41.8%)	0.39

*Note*: Numbers are *n* (%) or mean (standard deviation). Maternal outcomes reported per patient eligible for inclusion in the study. Perinatal outcomes reported for all pregnancies within the study period.

Both groups had similar rates of pregnancy loss at less than 14 weeks gestation, preterm birth, and cesarean delivery. The cesarean delivery rate for both groups was higher than the national average at 44.5% in the mechanical heart valve group and 41.8% in the bioprosthetic valve group (*p* = 0.39). The average gestational age at delivery was approximately 37 weeks for both groups (Table 2).

Among patients with a mechanical heart valve, 293 (76.3%) were reported to have used LMWH for anticoagulation, and 246 (64.1%) were reported to have used warfarin (Table 3) during pregnancy. Rates of maternal arrhythmia, heart failure, myocardial infarction, and stroke were similar between patients using LMWH and warfarin in pregnancy. However, the rate of endocarditis was much higher in patients receiving LMWH compared to warfarin (17.4% vs. 8.9%, *p* < 0.01). Perinatal outcomes including the rate of pregnancy loss at <14 weeks gestation, preterm birth, average gestational age at the time of delivery, and rate of cesarean delivery were similar between groups using LMWH and warfarin for mechanical valves (Table 3).

**TABLE 3:** Maternal and perinatal outcomes in patients with mechanical heart valves in pregnancy stratified by anticoagulation type.

	Low molecular weight heparin (*n* = 293)	Warfarin (*n* = 246)	*p* value
Maternal outcomes			
Arrhythmia	36 (12.3%)	31 (12.6%)	0.91
Heart failure	92 (31.4%)	71 (28.9%)	0.52
Endocarditis	51 (17.4%)	22 (8.9%)	**0.004**
Thromboembolism	<10	<10	–
Valve thrombosis	<10	<10	–
Myocardial infarction	14 (4.8%)	16 (6.5%)	0.38
Stroke	38 (13.0%)	42 (17.1%)	0.18
Preeclampsia/Severe preeclampsia	11 (3.8%)	<10	–
Postpartum hemorrhage	35 (11.9%)	<10	–
Perinatal outcomes			
Term birth	106 (36.2%)	78 (31.7%)	0.28
Preterm birth	84 (28.7%)	70 (28.5%)	0.96
Early pregnancy loss (<14 weeks)	48 (16.4%)	42 (17.1%)	0.83
Gestational age at delivery (weeks)	36.0 (±3.3)	35.7 (±3.3)	0.29
Cesarean delivery^a^	114 (60.0%)	86 (58.1%)	0.73

*Note*: Numbers are *n* (%) or mean (standard deviation). Maternal outcomes reported per patient eligible for inclusion in the study. Perinatal outcomes reported for all pregnancies within the study period.

^a^ Percentage is based on number of term and preterm births.

## 4 DISCUSSION

This is a large contemporary retrospective cohort study comparing pregnant patients with mechanical and bioprosthetic valves in pregnancy. We identified slightly more favorable maternal and perinatal outcomes among patients with biologic valves compared to mechanical heart valves.

Our study demonstrates that patients with a mechanical heart valve are at risk of several adverse outcomes in pregnancy, including heart failure and stroke. Patients with a mechanical heart valve in pregnancy are also at risk of developing an arrhythmia, endocarditis, and myocardial infarction, though the rates of these events were not significantly higher when compared to patients with bioprosthetic valves in pregnancy.

The rate of heart failure was much higher in this cohort compared to previous studies on patients with prosthetic valves in pregnancy. In one retrospective cohort study based on administrative data from California, New Jersey, and New York State between 1990 and 2015 evaluating patients with mechanical and biologic heart valves in pregnancy, the incidence of acute cardiac events (acute heart failure, cardiac arrest/shock, ventricular arrhythmia, and pulmonary edema) in pregnancy was low at <3%, with no difference between patients with mechanical and biologic valves [6]. However, 43%–56% of pregnant patients in this study had congestive heart failure at baseline. Additionally, the rate of hypertension among patients in this study was lower (15%–21%) compared to the patients in our study (62%), and hypertensive disorders are known to be associated with maternal cardiomyopathy [10, 11]. In the outcomes data from The Registry of Pregnancy and Cardiac disease III (ROPAC III) published in 2025, the rate of heart failure in patients with a mechanical valve was found to be 5.9%, while in patients with a biological valve it was found to be 7.1% [3], both of which are at least three times lower than the rate observed in this study. It is unclear what could be driving this difference in the rate of heart failure in our study compared to ROPAC III, though this may be at least partially due to inaccurate characterization or coding of maternal cardiac outcomes. Additionally, the patients in our study with mechanical valves had much higher rates of chronic hypertension (62%) compared to those in ROPAC III (4.4%).

The higher rate of cerebrovascular events/stroke observed in patients with mechanical valves compared to prosthetic valves in this study is consistent with previous studies [12]. However, the rate of stroke observed in this study (11.7%) was much higher than that seen in the ROPAC registry in 2015 (1.4%) [7]. This could be due to the higher rate of medical comorbidities in the population included in this study, such as the increased presence of chronic hypertension and smoking. The incidence of thromboembolic events and valve thrombosis in this cohort (<3%) was similar to that seen in some studies of patients with mechanical and bioprosthetic valves in pregnancy [6, 12], but was lower compared to that observed in others [3, 7]. The incidence of preeclampsia observed in this cohort was similar to the incidence observed in previous studies in patients with mechanical and biologic valves [3].

In patients with pregnancies affected by mechanical heart valves, outcomes were similar for patients who were using LMWH and warfarin with the exception of postpartum hemorrhage, which is consistent with previous studies on anticoagulation in pregnancy, suggesting that both may be reasonable options [8].

The modified World Health Organization (mWHO) classification system for maternal cardiovascular disease in pregnancy, which is a validated risk assessment tool for pregnant patients with cardiac disease, classifies mechanical valves in pregnancy as mWHO Class III. This classification suggests a significantly increased risk of severe maternal morbidity and mortality [13]. The data presented here, which identified high rates of maternal heart failure, arrhythmia, and stroke in patients with mechanical valves in pregnancy, support the current designation of mWHO Class III for this population.

### 4.1 Strengths and limitations

This study's strengths include the large sample size of a rare clinical entity in pregnancy. However, this study has several limitations. First, this was a retrospective study that relied on deidentified coding and billing data. For this reason, clinical characteristics and outcomes could not be manually confirmed. While Epic Cosmos has been shown to include a diverse population that is representative of the United States population, our cohort is predominantly White Non‐Hispanic. This may reflect a lack of diversity in the participating medical centers. Therefore, these data may not be generalizable to other populations. Given that Epic Cosmos provides access to aggregate‐level data, it was not possible to adjust for confounders using regression analysis, and only two‐sided statistical tests could be performed. For this reason, several clinical outcomes assessed, such as the rates of heart failure or stroke, could have been overestimated in this study cohort. For this reason, this study should be considered descriptive. Additionally, the presence or absence of valvular dysfunction in the study cohort could not be determined, nor could medication doses, which could have influenced the results of this study. Finally, we are unable to identify the exact timing of cardiac events in pregnancy related to trimester or immediate postpartum period with the current data set, and given that only aggregate‐level data were available, no adjusted analyses could be performed.

## 5 CONCLUSION

Our findings support the current designation of mWHO Class III for mechanical heart valves in pregnancy given the high rates of cardiac morbidity seen in this population. The presence of a mechanical heart valve in pregnancy is associated with adverse cardiac outcomes including heart failure, arrhythmia, and stroke. Many of these risks exist for patients with bioprosthetic heart valves as well. Perinatal outcomes may be similar for patients using LMWH and warfarin in pregnancy in the setting of a mechanical heart valve. Future larger studies are needed to replicate this work and should focus on the optimal timing and dosage of anticoagulation strategies in patients with mechanical heart valves in an effort to improve maternal outcomes.

## AUTHOR CONTRIBUTIONS

**Angela Essa**: Conceptualization; investigation; writing—original draft; writing—review and editing; methodology; visualization. **Lara C. Kovell**: Conceptualization; investigation; writing—review and editing; supervision; methodology. **Gianna L. Wilkie**: Conceptualization; investigation; writing—review and editing; methodology; formal analysis; supervision; visualization; data curation.

## CONFLICT OF INTEREST STATEMENT

The authors declare no conflicts of interest.

## FUNDING INFORMATION

The authors received no specific funding for this work.

## ETHICAL APPROVAL

This study was deemed exempt by the University of Massachusetts Chan Medical School Institutional Review Board given all data are deidentified.

## Data Availability

The data and statistical tools used for this research study will be made available upon request.
